# Does the Heel’s Dissipative Energetic Behavior Affect Its Thermodynamic Responses During Walking?

**DOI:** 10.3389/fbioe.2022.908725

**Published:** 2022-06-27

**Authors:** Nikolaos Papachatzis, Dustin R. Slivka, Iraklis I. Pipinos, Kendra K. Schmid, Kota Z. Takahashi

**Affiliations:** ^1^ Department of Biomechanics, University of Nebraska at Omaha, Omaha, NE, United States; ^2^ School of Health and Kinesiology, University of Nebraska at Omaha, Omaha, NE, United States; ^3^ Department of Surgery, University of Nebraska Medical Center, Omaha, NE, United States; ^4^ Department of Biostatistics, University of Nebraska Medical Center, Omaha, NE, United States

**Keywords:** foot, heel-strike, temperature, negative-work, locomotion, collision, energetics, biothermomechanics

## Abstract

Most of the terrestrial legged locomotion gaits, like human walking, necessitate energy dissipation upon ground collision. In humans, the heel mostly performs net-negative work during collisions, and it is currently unclear how it dissipates that energy. Based on the laws of thermodynamics, one possibility is that the net-negative collision work may be dissipated as heat. If supported, such a finding would inform the thermoregulation capacity of human feet, which may have implications for understanding foot complications and tissue damage. Here, we examined the correlation between energy dissipation and thermal responses by experimentally increasing the heel’s collisional forces. Twenty healthy young adults walked overground on force plates and for 10 min on a treadmill (both at 1.25 ms^−1^) while wearing a vest with three different levels of added mass (+0%, +15%, & +30% of their body mass). We estimated the heel’s work using a unified deformable segment analysis during overground walking. We measured the heel’s temperature immediately before and after each treadmill trial. We hypothesized that the heel’s temperature and net-negative work would increase when walking with added mass, and the temperature change is correlated with the increased net-negative work. We found that walking with +30% added mass significantly increased the heel’s temperature change by 0.72 ± 1.91  ℃ (*p* = 0.009) and the magnitude of net-negative work (extrapolated to 10 min of walking) by 326.94 ± 379.92 J (*p* = 0.005). However, we found no correlation between the heel’s net-negative work and temperature changes (*p* = 0.277). While this result refuted our second hypothesis, our findings likely demonstrate the heel’s dynamic thermoregulatory capacity. If all the negative work were dissipated as heat, we would expect excessive skin temperature elevation during prolonged walking, which may cause skin complications. Therefore, our results likely indicate that various heat dissipation mechanisms control the heel’s thermodynamic responses, which may protect the health and integrity of the surrounding tissue. Also, our results indicate that additional mechanical factors, besides energy dissipation, explain the heel’s temperature rise. Therefore, future experiments may explore alternative factors affecting thermodynamic responses, including mechanical (e.g., sound & shear-stress) and physiological mechanisms (e.g., sweating, local metabolic rate, & blood flow).

## Introduction

The majority of the terrestrial legged locomotion gaits, like human walking, necessitate the dissipation of impact forces when the feet collide with the ground (Schmitt and Larson, 1995; Ruina et al., 2005; Lee et al., 2013; Miao et al., 2017; Usherwood, 2020; Zeininger et al., 2020). For instance, a large portion of energy dissipation (i.e., net-negative work) occurs immediately after the collision of the human heel with the ground. (Gefen et al., 2001; Chi and Schmitt, 2005; Takahashi et al., 2017; Baines et al., 2018; Honert and Zelik, 2019; Papachatzis et al., 2020). Such net-negative collision work is a vital feature of walking as the legs transition from one step to the next (Donelan et al., 2002a; Donelan et al., 2002b; Kuo, 2002, 2007; Kuo et al., 2005; Kuo and Donelan, 2010). Additionally, energy dissipation during the heel collision may highlight the heel’s protective mechanism to absorb impact and minimize trauma or injuries (Pain and Challis, 2001; Wearing et al., 2014). However, despite the importance of collisional energy losses during heel-strike, there remains a paucity of evidence on how the human heel dissipates (i.e., loses) mechanical energy. Consequently, a study of human bipedal walking is incomplete without understanding how the heel dissipates and utilizes net-negative work during heel-strike.

Conservation of energy suggests energy is a quantity that cannot be lost or created out of nothing. Therefore, the dissipated kinetic energy should be converted to heat and/or sound when the heel collides with the ground. Indeed, previous experiments have established that the collision between the heel and the ground produces an acoustic impact (Li et al., 1991; Ekimov and Sabatier, 2006; Visell et al., 2009; Larsson, 2014; Hung Au et al., 2021). Furthermore, principles of acoustics suggest that the amplitude of the produced sound should be proportional to the amplitude of the impact pressure. However, Hung Au et al., 2021 reported a statistically non-significant association between impact sound amplitude and vertical impact rate during running, with heel-strike running having the lowest peak sound amplitude (Hung Au et al., 2021). Thus, although energy dissipation as sound is plausible, it seems reasonable to predict that the heel’s collision energy during walking may be dissipated as heat.

Indeed, the principles of thermodynamics suggest that the heel’s net-negative collision work could be converted into heat and cause a temperature increase. *In-vivo* and *in-vitro* studies have shown that the heel exhibits viscoelastic behavior under vertical compression with energy presumably dissipated as heat (i.e., hysteresis) between the compressive loading-unloading cycles (Bennett and Ker, 1990; Aerts et al., 1995; Pain and Challis, 2001; Ledoux and Blevins, 2007; Pai and Ledoux, 2011; Behforootan et al., 2017). Energy dissipation as heat accords with previous data, which showed that the foot’s sites, including the heel, increase their skin temperature even by ∼5.0 ℃ after 30 min of walking (Reddy et al., 2017). The first law of thermodynamics:

$$\Delta E_{heel}=Q_{heel}-W_{heel}$$ (1)

gives a relationship between the generation or uptake of heat ( $Q_{heel})$ , the production or absorption of mechanical work done $\left(W_{heel}\right)$ , and the change of internal energy $\left(\Delta E_{heel}\right)$ of the heel (sum of all kinetic and potential energy). Since each step of walking could be considered a cyclic process in which the volume of the heel periodically returns to its initial state, the internal energy change in one step cycle should be zero; that is, $\Delta E_{heel}=0$ . From the first law, we see that:

$$Q_{heel}=W_{heel}$$ (2)

Therefore, the temperature change of the heel $\left(\Delta T_{heel};℃\;\right)$ can be estimated by solving the specific heat equation:

$$\Delta T_{heel}=\frac{Q_{heel}}{mc}$$ (3)

Since a unit change in Kelvin (K) equals a unit change in Celsius (°C), the SI units are (°C).

Inserting the Eq. 2 into Eq. 3 yields:

$$\Delta T_{heel}=\frac{W_{heel}}{mc}$$ (3a)

where $W_{heel}$ is the amount of net-negative work dissipated by the heel (kJ) , m is the mass of the heel (kg) and c is the specific heat of the heel’s tissues: blood: 3.84 (kJkgK) , muscle:  3.80(kJkgK), skin: 3.39 (kJkgK), fat: 2.30(kJkgK) and bone: 1.26−1.30(kJkgK) (Giering et al., 1996; Feldmann et al., 2018).

For a given specific heat capacity c , and a given heel mass m , Eq. 3a allows us to make theoretical predictions of the heel’s temperature increase during walking, in the absence of other heat regulation mechanisms (e.g., sweating, blood circulation), through estimates of the specific heat capacity of tissues (Giering et al., 1996; Feldmann et al., 2018) and mechanical work data of the heel during walking (Papachatzis et al., 2020). To do so, we assume that the composition of the heel is homogenous, somewhere between whole blood (upper bound for specific heat) or whole bone (lower bound for specific heat), and that the mass of the heel is roughly 1/3 of the foot’s mass (Bruening et al., 2012) (roughly ∼0.4 kg for an 83 kg individual; Plagenhoef et al., 1983). The structures surrounding the heel of an 83 kg healthy human lose ∼3 J of energy per step immediately after the heel strike (Papachatzis et al., 2020), which extrapolates to ∼1.8 kJ of energy during 10 min of walking (assuming ∼1 Hz step rate). Under these assumptions, Eq. 3a would predict a lower bound and upper bound temperature change between 1.22 and 3.7 (℃) , respectively. These values are similar to previously reported *in-vivo* heel temperature (∼2.3 ℃ ) after 10 min of walking (Reddy et al., 2017), despite the presence of other thermoregulation mechanisms (e.g., sweating, blood circulation). Thus, these predictions could suggest that net-negative work may potentially explain the heel’s temperature increase observed in previous walking studies (Hall et al., 2004; Yavuz et al., 2014; Reddy et al., 2017; Gonzalez et al., 2021).

If indeed the heel’s energy is dissipated as heat, such knowledge would be a valuable tool for scientists studying the feet and legs of various species. For example, it could provide a foundation to study how mechanical energy of the legs and feet is converted or utilized by the body during terrestrial locomotion. In humans, such knowledge could allow us to understand factors that influence heel temperature regulation, leading to new insights into the prevention of foot skin complications, such as blisters, calluses, and ulcers which can be particularly unfavorable in patients with diabetes and peripheral artery disease (Armstrong et al., 2007; Maddah and Beigzadeh, 2020; van Netten et al., 2020; Chatzistergos et al., 2021).

To the best of our knowledge, no *in-vivo* studies have directly examined if the human heel dissipates net-negative mechanical work as heat. Therefore, this study examined the relationship between the heel’s mechanical energy and thermal responses during walking. We experimentally increased the human heel’s collision forces (*via* walking with added mass) (Papachatzis et al., 2020) and investigated its effect on the heel’s thermodynamic responses. We hypothesized that when participants walk with higher added body mass levels, the magnitudes of heel’s temperature change and net-negative work (i.e., energy dissipated) will increase. Additionally, we hypothesized that an increase in temperature is correlated with higher magnitudes of energy dissipated as net-negative work by the heel.

## Materials and Methods

### Participants

Twenty healthy young adults volunteered to participate in this research study (5 females, 15 males; age = 24.4 ± 2.8 years; height = 1.74 ± 0.07 m, mass = 83.6 ± 21.2 kg; means ± standard deviation). The sample size was based on a power analysis using published data comparing foot temperature differences between multiple walking cadences (Reddy et al., 2017). For an effect size of 0.81, a sample size of 19 participants will provide 80% power to detect comparable differences, with significance set to *α* = 0.016. Participants were free of cardiac and neurological pathologies (such as arrhythmia, heart attack, and stroke) and any musculoskeletal or pathological problems (such as osteoarthritis, bone fractures, etc.). The Institutional Review Board at the University of Nebraska Medical Center approved the experimental protocol, and all participants signed an informed consent form before participating in the experimental protocol.

### Experimental Protocol

**FIGURE 1:**
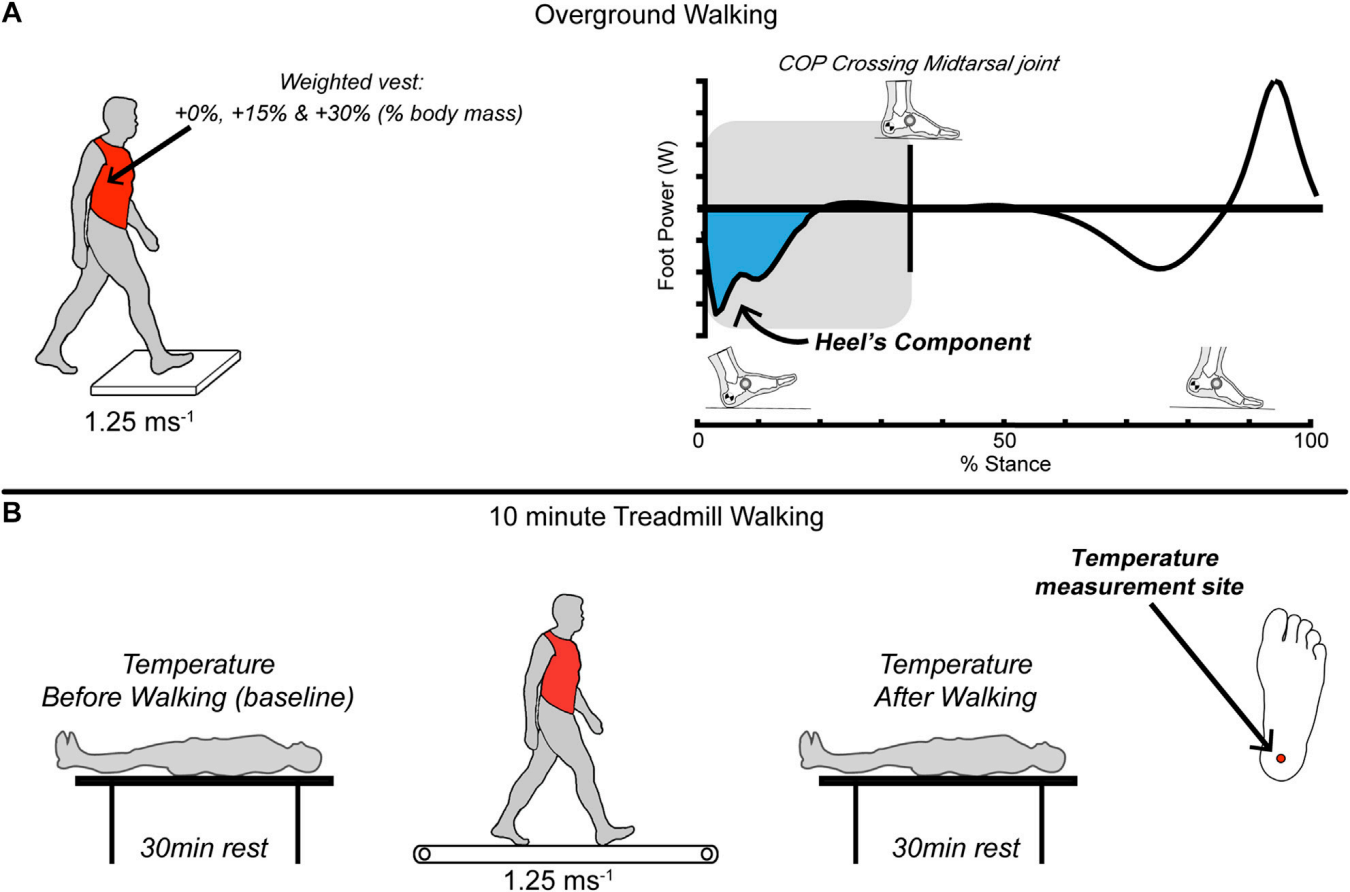
A total of 20 healthy young adults (N = 20) completed two barefoot randomized walking protocols: **(A)** overground on force plates and **(B)** 10 min on a treadmill (both at 1.25 m s^−1^). Participants carried (*via* weight vest) three different randomized levels of symmetrical loads: +0%; no added body mass, +15%, and +30% of their body mass. We used the overground walking trials to collect foot mechanics data (kinematic & kinetic), whereas the treadmill trials measured the foot temperature data. We quantified the mechanical power and work done by the foot using a unified-deformable analysis. We computed the work when the center-of-pressure was underneath the heel segment during the early stance phase to isolate the heel contribution. An estimate of the total work over the 10 min of treadmill walking was calculated by multiplying the average work per step measured in overground trials by the number of steps taken in 10 min of treadmill walking. Temperature measurements were taken immediately before and after each treadmill trial at the bottom of the right foot, including the heel pad. We computed the change in temperature of the heel before and after each walking trial.

The participants walked barefoot over the ground and on a treadmill (10 min per trial) while carrying three different levels of added mass: +0; no added body mass, +15%, and +30% relative to their body mass. We chose the level of added mass comparable to previous studies (Griffin et al., 2003; Silder et al., 2013; Huang and Kuo, 2014; Mooney et al., 2014; Rice et al., 2017) and considered safety risks. We used the overground walking trials to collect foot mechanics data, whereas the treadmill trials measured the foot temperature data (Figure 1). The walking speed was controlled at 1.25 m s^−1^ for overground and treadmill walking. We monitored the speed using timing gates (Dashr Elite Kit, Lincoln, NE, United States) and verbal feedback for overground walking. The overground versus treadmill trials were randomized, and the order of the added mass trials within the overground/treadmill trials was also randomized.

Participants carried the added mass wearing a weighted vest (M.I.R. Pro Weighted Vest, San Jose, CA, United States). We used a weighted vest because, compared to other methods, the symmetrical distribution of the carrying loads reduces the muscular activity and postural sway and has a smaller effect on the anterior-posterior center-of-mass location (Datta and Ramanathan, 1971; Bobet and Norman, 1984; Simpson et al., 2012; Sahli et al., 2013; Liew et al., 2016).

### Analysis: Foot and Heel Mechanics (Overground Walking)

The overground walking trials were performed over a ∼10 m long walkway consisting of five force plates aligned serially (AMTI Inc., Watertown, MA, United States), and ground reaction force data were collected at 1,080 Hz. We placed retro-reflective markers on bony landmarks of the feet, ankles, and knees while we used clusters to track the movement of the shank, thigh, and pelvis. We selected the landmarks based on Bruening et al. (2012), and we defined three segments within the foot (hindfoot, midfoot, and hallux). We used an eight-in an eight-infrared camera motion analysis system (Raptor-4S, Motion Analysis Corp., Mountain View, CA, United States) to capture the position of the retro-reflective markers on the lower extremity relative to the global reference system of the laboratory at 180 Hz. To minimize the error in the center of pressure (C.O.P.) estimates, we used the CalTester tool to assess the accuracy of the force platform when used in conjunction with the motion capture system (Holden et al., 2003), where we found an average error of 2.04 ± 1.35, 1.34 ± 1.23 and 1.36 ± 0.91 mm (means ± standard deviation of the five force plates) for the three axes.

We processed only the clean stance phases when the entire foot came in contact within the force plate’s borders without overlapping adjacent force plates or the floor. We analyzed approximately five stance phase data series (e.g., 3–7 per participant) for each walking trial for each participant. We averaged the stance data of each participant for each variable (e.g., negative, positive, and net work). We filtered the raw data by applying a second-order dual-pass low-pass Butterworth filter of 6 Hz for kinematic data and 25 Hz for kinetic data. A 20 N threshold for the vertical ground reaction force defined the start and the end time for each stance phase of walking. We collected, processed, and analyzed the data using Cortex motion analysis software (Motion Analysis Corp.), Visual3D (C-motion, Germantown, MD, United States), and MATLAB R2021a (MathWorks, Natick, MA, United States).

We quantified the mechanical power and work contribution (i.e., energy dissipation, absorption, return or generation) of the heel’s surrounding structures (e.g., fat pad) using computational methods described in our previous work (Papachatzis et al., 2020). Briefly, we quantified the mechanical power and work of all the structures distal to the hindfoot’s center of mass (i.e., structures of the entire foot) using a unified deformable segment analysis (Takahashi et al., 2017). Then, we determined the timing in which the C.O.P. crossed anteriorly to the midtarsal joint in the laboratory coordinate system. Based on a prior study (Bruening and Takahashi, 2018), we expected that the work performed when the C.O.P. was posterior to the midtarsal joint (i.e., C.O.P. was underneath the hindfoot segment) would include mostly contributions from structures underneath the hindfoot (e.g., heel pad).

For each stance phase, we integrated the mechanical power with respect to time to compute mechanical work (positive, negative, and net). To estimate work done by the foot during 10 min of walking, we multiplied the work per step computed from the overground trials by the number of steps estimated from the treadmill data (*via* the average step frequency observed). The stance phase was detected from the treadmill trials using a kinematics-based event detection algorithm using the heel and toe markers (Zeni et al., 2008).

### Analysis: Foot and Heel Temperature (Treadmill Walking)

Heel pad temperature measurements were obtained from the right foot immediately before and after each treadmill trial (10 min) using a type T cooper thermocouple skin-surface probe (SST-2, Physitemp, Instruments, Clifton, NJ, United States) with an accuracy of ±0.1 (°C) connected to a 4-channel Extech SDL200 data logger thermometer with a sampling rate of 3,600 samples per second (Extech SDL200, Extech Instruments, Waltham, MA, United States). To ensure stable baseline temperature measurements before each added mass trial, we allowed an acclimatization period to adjust the feet of each participant to room temperature. Briefly, participants lay quietly on the treatment table for 30 min before each treadmill trial. At the start of this 30 min, 70% isopropyl alcohol was sprayed over the right foot, shank, and thigh, and 70% isopropyl alcohol wipes were used to disinfect the foot. We defined “baseline temperature” as the temperature of the recording site after the acclimatization period. All measurements were obtained while the subject was lying down on a standard treatment table.

While the focus of this study was quantifying heel pad temperature, we obtained additional temperature measurements from different sites of the plantar and dorsal surface of the foot and the anterior-posterior aspect of the shank and thigh. Specifically, we measured the temperature of the inferior aspect of the hallux, first and fifth metatarsal heads, the superior aspect of the first and fifth metatarsal heads; the temperature over the tibialis anterior muscle belly, posteriorly over the gastrocnemius medialis muscle belly, anteriorly over the vastus lateralis muscle belly and posteriorly over the semimembranosus muscle belly. Also, we measured the absolute tympanic temperature without any adjustments using a tympanic thermometer, placing it in the participant’s right ear. The thermometer had an accuracy of ±0.1 (°C), range (33.0–42.0°C), and recorded the temperature after ∼1 s (Covidien Genius 2, Cardinal Health, Dublin, Ireland). In addition, we measured the temperature of the treadmill belt surface by placing two contact thermometer probes on the treadmill’s surface in areas where the participants frequently stepped.

The probes of the contact thermometer were placed over the specific regions of interest until a stable measurement was obtained (∼20–30 s per site). Two investigators used the four probes simultaneously to minimize the recording time. The order of temperature measurement was as follows: all four plantar foot sites first, the two dorsal foot sites and two anterior sites (shank and thigh) second, the two posterior sites (shank and thigh) and core temperature third, and the two treadmill sites last. We also recorded the laboratory’s temperature and humidity at the end of the above temperature measurements. For all the treadmill walking trials, the participants had two reflective markers placed on each foot, one over the Achilles tendon insertion into the calcaneus and one on top of the hallucis over the nailbed.

We computed the change in temperature (ΔT; ℃) across each measurement site as the difference between the temperature before and after each treadmill walking trial.

### Supplementary Experiment: Heel Temperature Measurements Validation

We performed an additional experiment to examine the validity of the heel temperature measurements acquired from the treadmill trials (see Supplementary Material). For this experiment, 12 participants walked barefoot for 10 min in a randomized order on a treadmill and on an overground track while carrying (*via* weight vest) two different levels of added mass: +0% (no added body mass) and +30% relative to their body mass. In addition, we controlled the walking speed for the treadmill and overground track trials at 1.25 m s^−1^. The order of the added mass levels was also randomized. We obtained all measurements precisely the same procedures described in the *Analysis: Foot and Heel Temperature (Treadmill Walking)* section.

### Statistical Analysis: Hypotheses Testing

To determine the effect of the added mass on the heel temperature change after 10 min of barefoot walking on 10 min of negative and net-negative extrapolated barefoot work (dependent variables), we used repeated-measures ANOVAs (three levels of added mass). In addition, we used the Simulate technique to adjust *p*-values for multiple comparisons when we detected significant effects.

We used linear mixed models to examine the correlation between the heel’s mechanical work (negative or net-negative work; two different models) and temperature change while accounting for the repeated measures among participants due to different levels of added mass.

### Statistical Analysis: Exploratory Analysis

We performed an exploratory analysis using linear mixed models to examine the correlation between the heel’s net-negative mechanical work extrapolated to 10 min of barefoot walking (predictor variables) and the heel’s temperature change (response variable). We used a manual variable backward selection procedure accounting for additional predictor variables: gender, body weight, height, foot length, impulse (vertical, mediolateral, and anterior-posterior) extrapolated to 10 min of walking, treadmill surface temperature after 10 min of walking, and heel temperature before the walking trials.

For all analyses, we used SAS version 9.4. The threshold for a significant effect was set to *p*-value ≤ 0.05, and residuals were examined to ensure model assumptions and fit.

## Results

### Effect of Added Body Mass on Heel’s Mechanical Work (Extrapolated to 10 min of Barefoot Walking)

**FIGURE 2:**
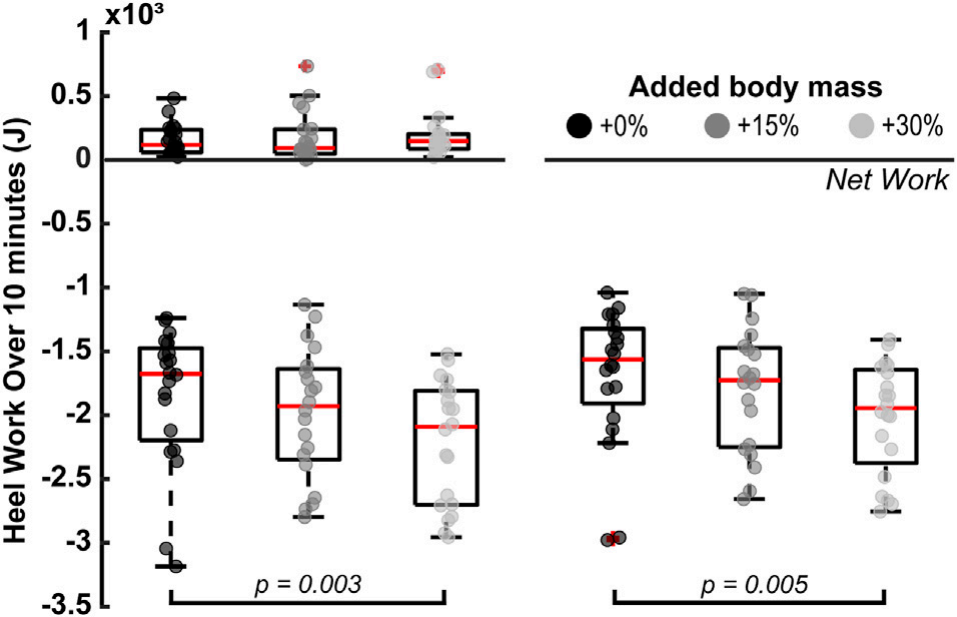
Walking with an additional 30% of body mass significantly increased the magnitudes of the extrapolated to 10 min, negative (*p* = 0.003) and net-negative (*p* = 0.005) work during the heel strike phase. The horizontal square brackets indicate the adjusted significant pair-wise comparisons (N = 20). We added jitter to the data on the x-axis only for visualization purposes, using the scatter function in MATLAB R2021a.

Added body mass had a statistically significant effect on the heel’s negative and net-negative mechanical work extrapolated 10 min of barefoot walking (*p* = 0.004 and *p* = 0.007 respectively; Figure 2). Specifically, the magnitude of negative work significantly increased by 364.43 ± 404.06 J between +0% and +30% added mass (*p* = 0.003), but not between +0% and +15% added mass (*p* = 0.400) or between +15% and +30% added mass (*p* = 0.077). Additionally, the magnitude of net-negative work significantly increased by 326.94 ± 379.92 J between +0% and +30% added mass (*p* = 0.005), but not between +0% and +15% added mass (*p* = 0.505) or between +15% and +30% added mass (*p* = 0.084).

### Effect of Added Body Mass on Heel’s Temperature Change After 10 min of Barefoot Walking

**FIGURE 3:**
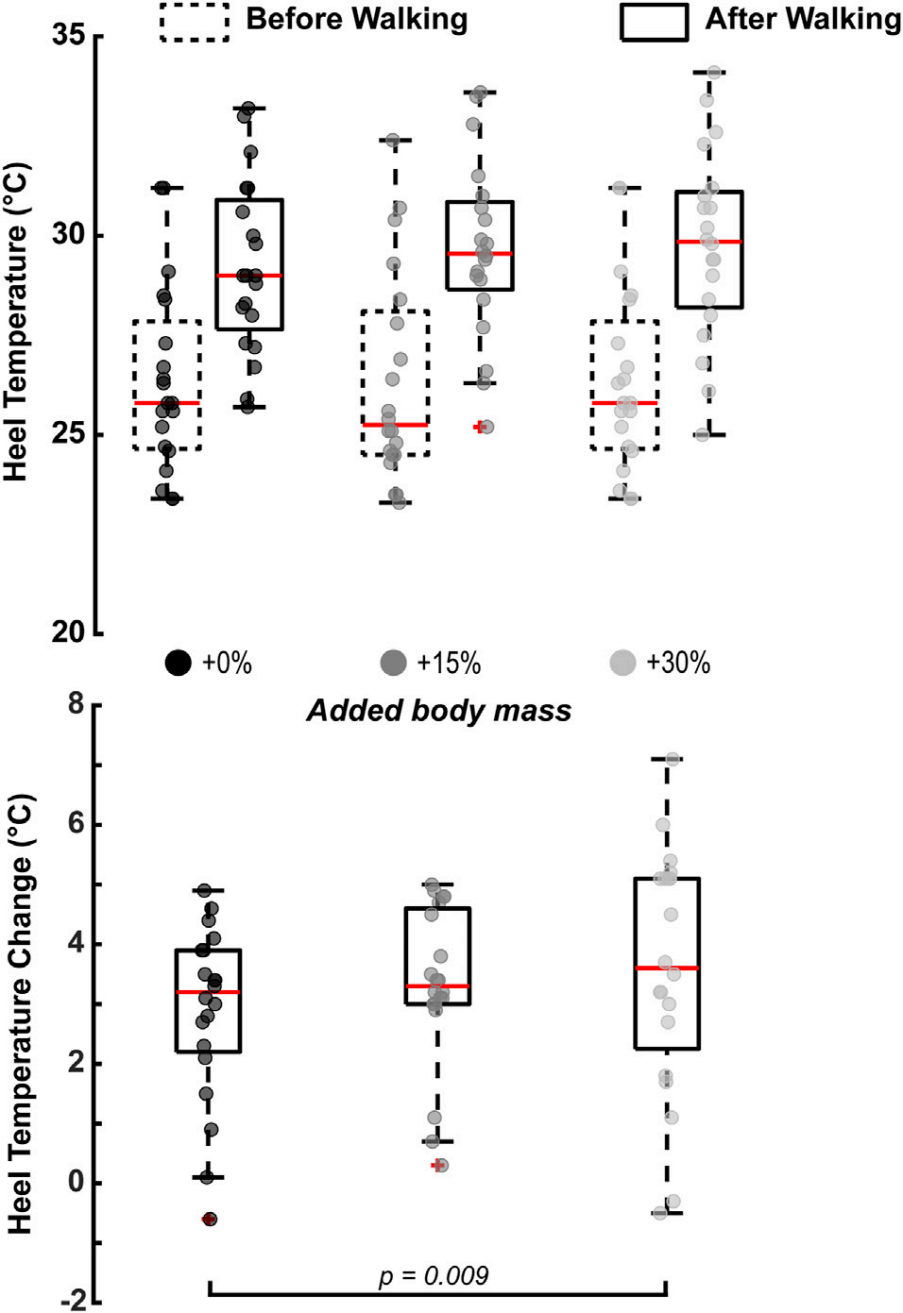
Walking with an additional 30% of body mass significantly increased the heel’s temperature change after 10 min of barefoot walking (*p* = 0.009). Heel’s temperature increased with the rest of the condition but was not statistically significant. The horizontal square brackets s indicate the adjusted significant pair-wise comparisons (N = 20). We added jitter to the data on the x-axis only for visualization purposes, using the scatter function in MATLAB R2021a.

Added body mass had a statistically significant effect on the heel’s temperature change (ΔT; ℃) after 10 min of barefoot walking (*p* = 0.012; Figure 3). Specifically, the temperature change significantly increased by 0.72 ± 1.91 (ΔT; ℃) between +0% and +30% added mass (*p* = 0.009), but not between +0% and +15% added mass (*p* = 0.186) or between +15% and +30% added mass (*p* = 0.377).

Walking with added mass had no statistical effect (*p* = 0.322) on the tympanic (body) temperature change (+0%: 0.02 ± 0.22°C, +15%: 0.005 ± 0.30°C, +30%: 0.55 ± 2.33°C; means ± standard deviation).

We also examined the influence of the treadmill on the heel temperature measurements by comparing the temperature measurements obtained from the treadmill trials with temperature measurements obtained from overground trials. The difference in the heel’s temperature change between the two added mass conditions (i.e., $\Delta T_{+30\text{\%}}-\Delta T_{+0\text{\%}})$ between overground and treadmill walking was not statistically different (*p* = 0.272; panel C; see Supplementary Material).

### Correlation Between Heel’s Temperature Change and Net-Negative Mechanical Work

There was no statistically significant correlation of negative or net-negative mechanical work extrapolated to 10 min of barefoot walking with heel’s temperature change after 10 min of barefoot walking (*p* = 0.383 and *p* = 0.277; respectively).

### Exploratory Analysis: Additional Variables That Could Explain Heel Temperature Change

We adjusted for the gender, body weight, height, foot length, impulse, treadmill surface temperature after 10 min of walking, and heel temperature before the walking trial. The model of the analysis showed that only the temperature of the heel before walking (i.e., baseline) (*p* < 0.001) had a statistically significant effect on the heel’s temperature increase after 10 min of barefoot walking (Supplementary Table S1). Furthermore, the heel temperature before walking had a negative slope, indicating that the temperature change was less for a higher temperature before waking.

## Discussion

**FIGURE 4:**
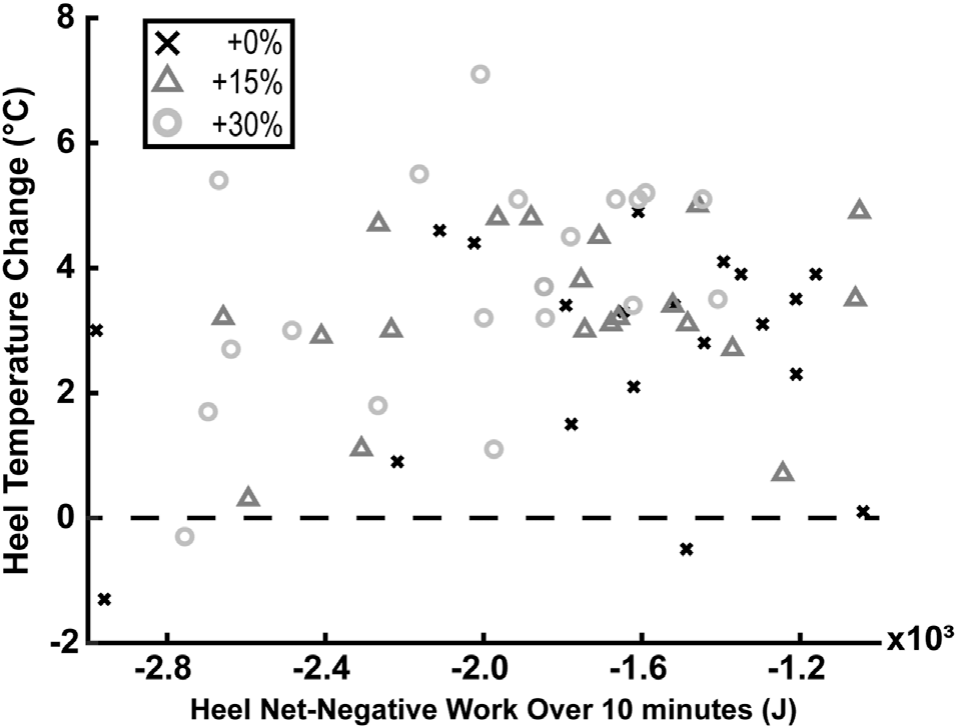
There was no statistically significant correlation between the heel’s net-negative mechanical work extrapolated to 10 min of barefoot walking and the heel’s temperature change after 10 min of barefoot walking (*p* = 0.277; N = 20). Values above the dashed line indicate that the temperature increased, while values below indicate the temperature decreased.

The collision between the leg and the ground is a vital feature for most terrestrial legged locomotion gaits, particularly human walking (Ruina et al., 2005; Hutchinson et al., 2011; Miao et al., 2017; Panagiotopoulou et al., 2019; Clemente et al., 2020; Zeininger et al., 2020) where the heel is often associated with performing net-negative work upon ground contact (Gefen et al., 2001; Takahashi et al., 2017; Baines et al., 2018; Honert and Zelik, 2019; Papachatzis et al., 2020). Here, we examined the relationship between the heel’s mechanical work and its thermodynamic responses by experimentally increasing the collisional forces during walking. Our results showed that walking for 10 min with +30% added mass increased the magnitude of net-negative work that the heel dissipates by 326.94 ± 379.92 J (*p* = 0.005; Figure 2) and increased the heel’s temperature change by 0.72 ± 1.91  ℃ (*p* = 0.009; Figure 3)—findings that confirmed our first hypothesis. These findings confirm prior studies suggesting heel’s dissipative behavior (Gefen et al., 2001; Takahashi et al., 2017; Baines et al., 2018; Honert and Zelik, 2019; Papachatzis et al., 2020) and that its temperature increases when walking for extended periods (Hall et al., 2004; Yavuz et al., 2014; Reddy et al., 2017). However, we found no significant correlation between the heel’s temperature increase and the magnitudes of net-negative work (*p* = 0.277; Figure 4)—a finding that refuted our second hypothesis.

Work from several groups has established that skin temperature fluctuations trigger thermoregulatory responses to maintain the temperature within a normal physiological range (Taylor et al., 1984; Rowell, 1990; Pérgola et al., 1994; Charkoudian, 2003; Crandall and González-Alonso, 2010). In the absence of such responses, the skin temperature would have elevated excessively. For instance, Reddy et al. (2017) found an inverse association between the foot’s baseline temperature and foot temperature change after walking (Reddy et al., 2017). This finding corroborates the model of our exploratory analysis, which suggests that higher baseline heel temperature (i.e., before walking) was associated with a smaller increase in temperature (*p* < 0.001; Supplementary Table S1). In our study, a few participants had drastically elevated heel skin baseline temperature than others (range 8.4  ℃ ) even though the order of the added mass walking trials had no significant effect on the baseline temperature (*p* = 0.318). It is possible that the elevated heel skin baseline temperatures had already activated the heat dissipation mechanisms like the vascular and sympathetic systems and prevented a harmful excessive temperature rise (Charkoudian, 2003). Another possibility is that the heel prevented an excessive temperature elevation by dissipating a portion of the collisional net-negative work as sound. While this seems unlikely based on a prior study (Hung Au et al., 2021), a direct measurement of sound is needed to verify or refute sound as a dissipation mechanism. An additional possibility is that the motion of the feet in the air, especially during the swing phase, could produce a cooling effect (Burton, 1934; Chang et al., 1987; Melnikov et al., 2020). Unfortunately, we did not collect sound amplitude or physiological measurements, like blood flow, local metabolic rate, or rate of sweating.

While we did not observe a correlation between net-negative work and the temperature of the heel, we found that the heel’s temperature change increased when walking with added mass by 0.72 ± 1.91  ℃ (*p* = 0.009; Figure 3). Therefore, our results indicate that additional factors, besides energy dissipation, explain the heel’s temperature rise. Prior studies have shown that the heel can experience shear forces during the collision (Stucke et al., 2012), and shear impulse (Gonzalez et al., 2021), and stress (Yavuz et al., 2014) are positively associated with the rise of plantar temperature after walking. Therefore, it is possible that walking with added mass induced greater shear stress on the heel (Jeong et al., 2021), deforming it repeatedly during the collision and explaining the heel’s temperature rise. However, when we accounted for the influence of the resultant shear impulse in our exploratory model, we found that the resultant shear impulse was not correlated with the temperature increase (*p* = 0.823). However, our experiment cannot conclusively refute the contribution of the shear impulse or stress to the heel’s temperature rise. Previous studies either have measured the association between shear and plantar temperature using custom-built pressure and shear plates (Stucke et al., 2012; Yavuz et al., 2014) or by experimentally manipulating the magnitudes of shear applied on foot by having the participants walk in circles with different radii (Gonzalez et al., 2021). Therefore, further research needs to combine measurements such as shear stress (Stucke et al., 2012; Yavuz et al., 2014) and *in-vivo* heel tissue mechanics (e.g., thickness, deformation, stiffness, hysteresis) (Liau et al., 2021) to understand their contribution to the heel’s skin temperature rise during walking.

Although we found an increase in the heel’s temperature during walking, this increase could be due to external heat stimuli, such as the treadmill surface. For instance, walking on a treadmill could have caused an irregular change in the heel’s temperature due to heating of the treadmill surface due to friction between the treadmill belt, the rollers, and the platform inside the treadmill’s deck. While our exploratory analysis suggested that the treadmill’s surface temperature had no significant effect on the heel’s temperature increase (*p* = 0.181; Supplementary Table S1), we performed an additional experiment to examine the validity of the heel’s temperature measurements acquired from the treadmill trials (see Supplementary Material). For this experiment, a subset of the participants walked barefoot for 10 min in a randomized order on a treadmill and on an overground track while carrying (*via* weight vest) two different levels of added mass: +0% (no added body mass) and +30% relative to their body mass. In addition, we controlled the walking speed for the treadmill and overground track trials at 1.25 m s^−1^. The supplementary experiment suggested that while the treadmill significantly affects the heel temperature measurements, the treadmill did not compromise our ability to detect temperature changes due to walking with added mass (Supplementary Figure S1). However, future experiments should consider the possible influence of the walking surface on the foot’s and heel’s temperature measurements. We also note that all of our participants walked barefoot. We chose barefoot walking because the mechanical characteristics of the shoe could influence our heel’s mechanical power and work calculations, which would have affected our ability to examine the mechanical-thermal relationships. Future studies should also consider the influence of footwear (e.g., shoes, orthoses, socks) on foot temperature responses during walking to increase the ecological validity.

In summary, our study revealed that the heel’s mechanical energy losses in healthy participants were not strongly correlated with the heel’s temperature changes. While this finding refuted our main hypothesis, it possibly highlights the agile control of the heel’s temperature by the vascular and sympathetic systems. Humans routinely perform tasks that require varying mechanical work demands during daily living, such as walking on slopes or stairs or continuously changing speeds. If all of the net-negative work done by the heel were dissipated as heat without additional thermoregulatory control mechanisms, performing basic daily activities may directly influence temperature increase, ultimately leading to skin complications, such as blisters and calluses. While our study involved healthy young participants, this study could be applicable for future studies that will examine impaired thermoregulation in individuals with neurovascular diseases (e.g., diabetes and peripheral artery disease) who are prone to foot complications (e.g., ulcers) (Armstrong et al., 2007; Maddah and Beigzadeh, 2020; van Netten et al., 2020; Chatzistergos et al., 2021). Our study provides a foundation to examine the link between mechanical energy, temperature responses, and the function of healthy and abnormal vascular and sympathetic systems in humans and/or animals. For example, future experiments may test alternative hypotheses related to energy dissipation mechanisms such as sound, shear forces, and skin blood flow, leading to novel insights for understanding how locomotion mechanics affect the thermoregulation of the foot. Thermoregulation is an essential homeostatic mechanism for mammals because most physiological processes include chemical reactions sensitive to temperature changes (Refinetti, 1999; Katschinski, 2004; Wooden and Walsberg, 2004). In the absence of such thermoregulation mechanisms, foot temperature could increase excessively during locomotor tasks, harming many terrestrial animals that have to cope with various terrains and environmental temperatures. Therefore, understanding the factors that influence healthy and dysfunctional thermoregulation will help us design new approaches for treating patients and identify new control mechanisms of terrestrial legged locomotion.

## Conclusion

Our results indicated no strong correlation between the heel’s net-negative work and temperature in healthy participants. This finding is likely related to agile temperature control of our feet to prevent excessive temperature elevations that may predispose the tissue to damage. Such temperature control is likely due to the vascular and sympathetic systems, which may be compromised in various vascular diseases such as diabetes. Future studies are needed to uncover further the mechanisms of temperature responses, which may lead to novel insights for understanding the causes of foot complications such as diabetic ulcers and identify new control mechanisms for terrestrial legged locomotion.

## Data Availability

The datasets presented in this study can be found in online repositories. The names of the repository/repositories and accession number(s) can be found below: https://doi.org/10.6084/m9.figshare.19450421.v2.
