# Supplementary material for: Does the Heel’s Dissipative Energetic Behavior Affect Its Thermodynamic Responses During Walking?
Source: Front Bioeng Biotechnol. 2022 Jun 27;10:908725. doi: 10.3389/fbioe.2022.908725 (PMC9271620; doi:10.3389/fbioe.2022.908725)
Supplement: Supplementary file 1 [file DataSheet1.pdf]

Supplementary Data:

Heel Temperature Measurements Validation: Comparing Treadmill & Overground Walking.

Rational

Walking on a treadmill can cause an irregular rise in heel temperature. That is because the friction between the running belt, the rollers, and the platform inside the treadmill's deck can cause the belt to heat up and transfer additional heat to the heel. This supplementary experiment aimed to examine the validity of the heel temperature measurements acquired from the treadmill trials. Therefore, we aimed to answer two questions; first, does the heel temperature increase after 10 minutes of barefoot overground walking with +30% of added body mass, and secondly, are the heel's temperature changes between overground and treadmill walking similar?

Methods

To achieve that, we recruited a subset of twelve participants (N=12, 7 females, 5 males; age = 23.7 ± 3.2 yrs; height = 1.68 ± 0.21 m, mass = 74.5 ± 17.4 kg; means ± standard deviation). One female participant's age, height, and mass are missing; therefore, means and standard deviations are calculated from N=11. Participants walked barefoot for 10 minutes in a randomized order on a treadmill and on an overground track while carrying (via weight vest) two different levels of added mass: +0% (no added body mass) and +30% relative to their body mass. The order of the added mass levels was also randomized. We controlled the walking speed for the treadmill and overground track trials at 1.25 m s^{-1} (the same speed as the treadmill and overground trials mentioned in the *Experimental Protocol* section). We controlled the walking speed for the overground track trials by dividing the track into four equal distance sections and providing verbal feedback about the time taken to walk each section. We obtained all measurements precisely the same procedures described in the *Analysis: Foot and Heel Temperature (Treadmill Walking)* section. In summary, the participants rested on a treatment table for 30minutes before each walking trial to allow the foot temperature to stabilize. We measured the before and after walking temperature for all walking trials at the same sites mentioned in the *Analysis: Foot and Heel Temperature (Treadmill Walking)* section. We computed temperature change (ΔT; ℃) as the difference between the temperature after and before each walking trial. Additionally, we computed the difference in temperature change between added mass conditions ($\Delta T_{\Delta T}$; ℃) as:

$$Overground\ \Delta T_{+30\%\ \text{added body mas}} - Overground\ \Delta T_{+0\%\ \text{added body mas}}$$

and

$$Treadmill\ \Delta T_{+30\%\ \text{added body mas}} - Treadmill\ \Delta T_{+0\%\ \text{added body mas}}$$

, where ΔT_{30} is the difference between the temperature after and before walking with +30% added mass and ΔT_0 is the difference between the temperature after and before walking with +0% added mass.

We used paired-samples t-tests to compare the heel's temperature changes between +0% and +30% added body mass overground walking and between +0% and +30% added body mass treadmill

walking. Also, we compared the difference in temperature change (i.e., $\Delta T_{+30\%} - \Delta T_{+0\%}$) between treadmill and overground walking. We analyzed the data using MATLAB R2021a (MathWorks, Natick, MA, USA). The significance level was considered as $p < 0.05$

Results and Discussion

We found that the heel temperature change increased significantly between +0% and +30% added mass during barefoot overground (p=0.014) and treadmill (p=0.021) walking (Sup. Figure 1; panel A. & panel B.; respectively). Additionally, the difference in the heel's temperature change (i.e., $\Delta T_{+30\%} - \Delta T_{+0\%}$) between overground and treadmill walking was not statistically different (p=0.272, Figure 1; panel C).

In conclusion, this experiment showed that 10 minutes of barefoot walking with +30% of added body mass increases the heel's temperature during overground and treadmill walking (Sup. Figure 1; panel A. & panel B.; respectively). Furthermore, the mechanical characteristics of the treadmill's moving parts (e.g., belt and rollers) affect to some extent but do not compromise the ability to detect temperature changes due to walking with added mass (Sup. Figure 1; panel C.). Overall, this supplementary experiment supports the validity of our main findings that the added mass affected heel's temperature, which was not explained by the artificial stimuli of the treadmill surface.

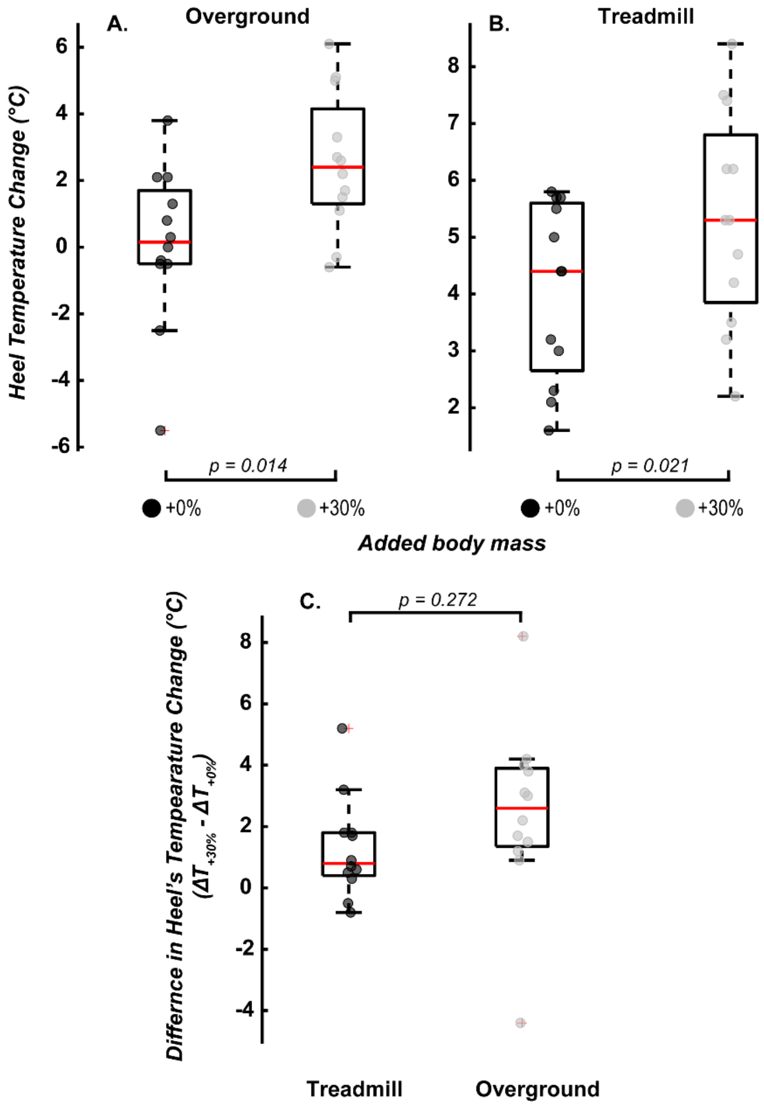

Sup. Figure 1: The differences in heel's temperature changes (i.e., $\Delta T_{+30\%} - \Delta T_{+0\%}$) between overground and treadmill walking were not statistically different (p=0.272; panel C.), supporting our main experiment's validity regarding heel temperature measurements. Walking with +30% added body mass significantly increased the heel's temperature change after 10 minutes of overground barefoot walking (p = 0.014; panel A.) and after 10 minutes of barefoot treadmill walking (p=0.021; panel B.). The horizontal square brackets indicate the adjusted significant pair-wise comparisons (N=12).

Sup. Figure 2: Average temperature measurements for additional anatomical sites obtained before and after the treadmill walking trial of the main experiment (N=20). The vertical error bars represent one standard deviation.

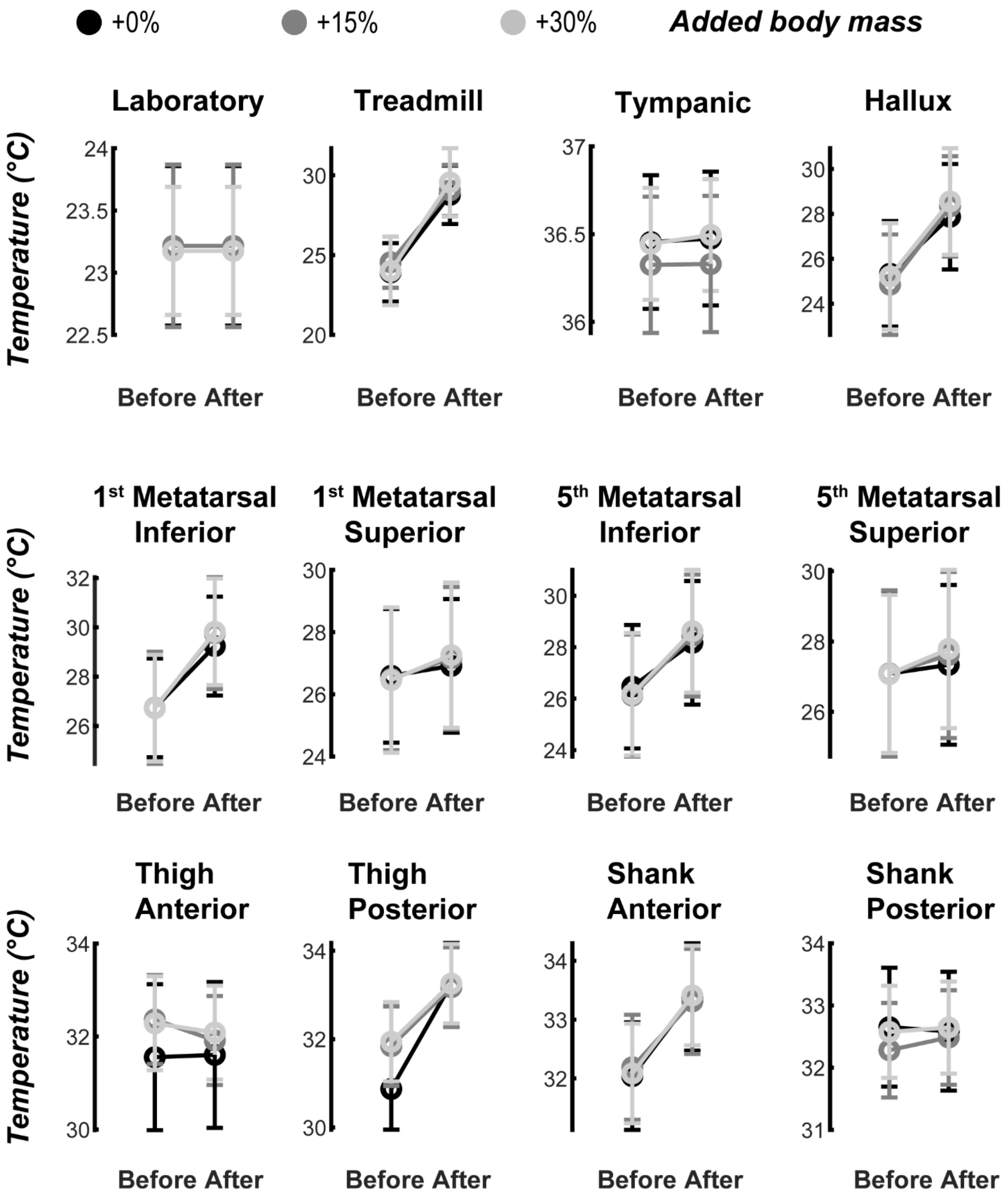

Sup. Figure 3: Heel temperature measurements immediately before and after each treadmill trial for all the participants of the main experiment (N=20).

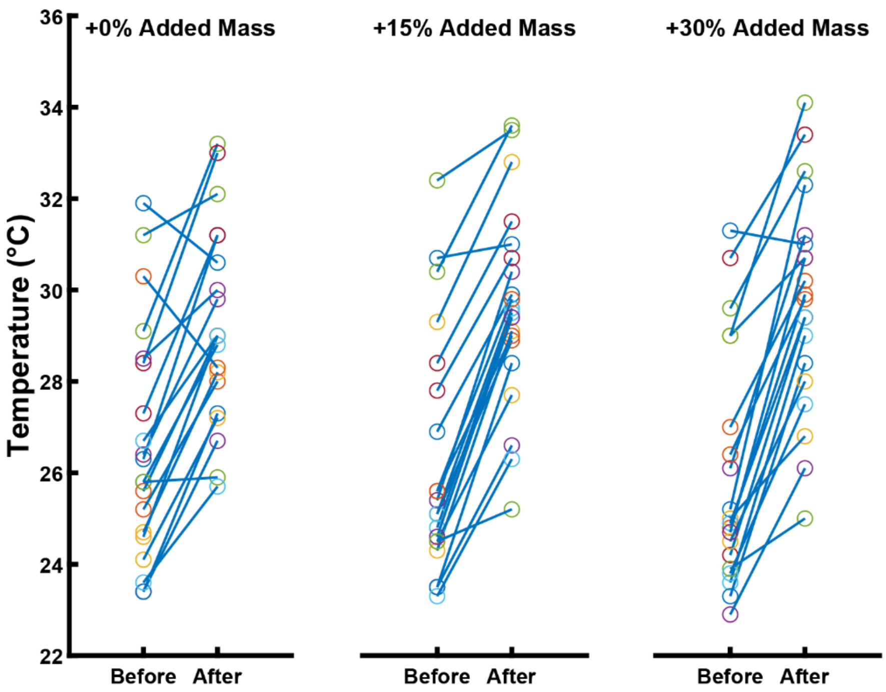

Sup. Table 1: We performed an explanatory analysis using a linear mixed model to examine the effect of additional variables on the heel temperature change for the data set of our main experiment (N=20).

Fixed Effects	Estimate	SE	t	p-value
Intercept	-0.905	15.646	-0.06	0.955
Gender (Females)	0.489	1.365	0.36	0.725
Gender (Males)	0	.	.	.
Weight (kg)	-0.014	0.026	-0.54	0.596
Height (cm)	0.027	0.073	0.37	0.717
Right Foot Length (cm)	0.263	0.389	0.68	0.509
Baseline Heel Temperature (°C)	-0.510	0.094	-5.42	<0.001
Treadmill Temperature After Walking (°C)	0.175	0.129	1.36	0.181
Absolute Vertical Heel Impulse (Ns)	0.00003	0.00003	1.02	0.313
Resultant Mediolateral and Anterior-posterior Heel Impulse (Ns)	0.00004	0.0002	0.23	0.823
Heel Net-negative Work extrapolated to 10min. of barefoot walking (J)	0.0005	0.0004	1.41	0.168
